# An outbreak of canine schistosomiasis in Utah: Acquisition of a new snail host (*Galba humilis*) by *Heterobilharzia americana*, a pathogenic parasite on the move

**DOI:** 10.1016/j.onehlt.2021.100280

**Published:** 2021-06-17

**Authors:** Eric S. Loker, Scott Z. Dolginow, Suzanne Pape, Colin D. Topper, Pilar Alda, Jean P. Pointier, Erika T. Ebbs, Melissa C. Sanchez, Guilherme G. Verocai, Randall J. DeJong, Sara V. Brant, Martina R. Laidemitt

**Affiliations:** a Center for Evolutionary and Theoretical Immunology, Museum of Southwestern Biology, Parasite Division, Department of Biology, University of New Mexico, Albuquerque, NM 87131, USA; b Mill Creek Animal Hospital, 125 E 300 S, Moab, UT 84532, USA; c 610 Dragonfly Trail, Moab, UT 84532, USA; d Centro de Recursos Naturales Renovables de la Zona Semiárida (CERZOS—CCT—CONICET Bahía Blanca), Camino de la Carrindanga km 7, Bahía Blanca 8000, Argentina; e PSL Research University USR 3278 CNRS-EPHE, CRIOBE, Université de Perpignan, France; f Department of Biology, Purchase College, State University of New York, Purchase, NY 10577, USA; g Department of Veterinary Pathobiology, College of Veterinary Medicine & Biomedical Sciences, Texas A&M University, College Station, TX 77843-4461, USA; h Department of Biology, Calvin University, 1726 Knollcrest Circle SE, Grand Rapids, MI 49546, USA

**Keywords:** Emerging infectious disease, Range expansion, Schistosomiasis, *Heterobilharzia*, *Galba humilis*, Vector biology

## Abstract

Parasites with complex life cycles engaging multiple host species living among different environments well-exemplify the value of a cross-cutting One Health approach to understanding fundamental concerns like disease emergence or spread. Here we provide new information regarding a pathogenic schistosome trematode parasite of both wild and domestic mammals that has recently expanded its known range from mesic/wet environments of the southeastern United States to the arid southwest. In 2018, 12 dogs living near a man-made pond in Moab, Utah, were found positive for *Heterobilharzia americana*, the most westerly report of this endemic North American schistosome, and the first from Utah. Raccoon scats collected near the pond were positive for *H. americana* eggs, and snails living near the pond's water line identified as *Galba humilis* shed *H. americana* cercariae, the first indication of natural infections in this widespread North American snail species. The susceptibility of *G. humilis* to *H. americana* was confirmed experimentally. Our studies support the existence of two variants of *H. americana* and emphasize the need for further investigations of lymnaeids and their compatibility with *H. americana,* to better define the future potential for its spread. Capture of a new species of intermediate host vector snail and construction of man-made habitats suitable for this snail have created the potential for a much more widespread animal health problem, especially for dogs and horses. *H. americana* will prove difficult to control because of the role of raccoons in maintaining transmission and the amphibious habits of the snail hosts of this pathogenic schistosome.

## Highlights

• The pathogenic canine schistosome *Heterobilharzia americana* expands its known range.
• First evidence of new snail vector, *Galba humilis.*
• *Galba humilis* is widely distributed in North America, enabling further spread.
• Raccoons are also important hosts and their increasing abundance also favors spread.

## Graphical abstract

**Figure:**
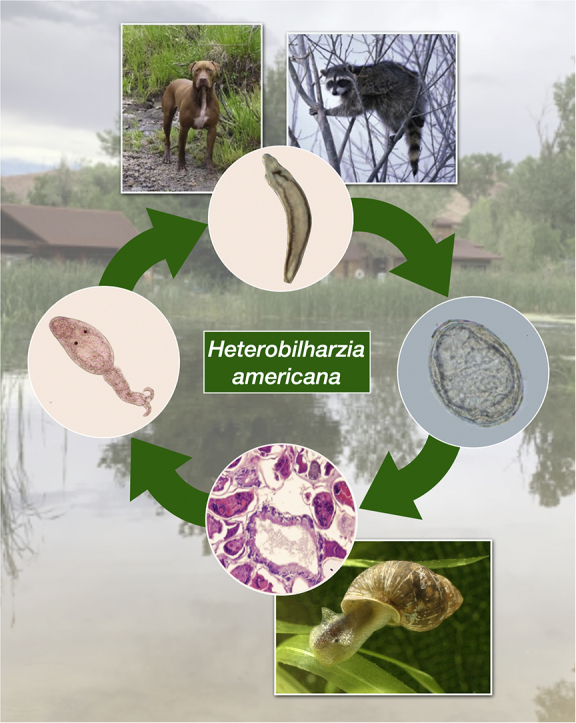

## 1 Introduction

The OneHealth concept recognizes that the health concerns of humans and wild and domestic animals and plants are intertwined and that changing environmental circumstances might have unforeseen effects leading to unexpected emergence of infectious diseases with far-reaching implications [1,2]. Along with the spillover of zoonotic pathogens like SARS-CoV-2 into new hosts [3], another factor driving infectious disease emergence is expansion of pathogens or their hosts or vectors into new environments. The range expansions of several tick species [4], and the meningeal worm *Parelaphostrongylus tenuis* [5] provide familiar examples. Here we discuss the expansion of an endemic pathogenic schistosome of wild and domestic mammals into new North America environments.

*Heterobilharzia americana* is one of two indigenous North American species of schistosome trematodes infecting mammals. It is a parasite of raccoons but is noteworthy for the broad range of other wild and domestic mammals it can infect, especially dogs [6]. In humans, cercariae of *H. americana* cause severe dermatitis but humans do not develop patent infections. However, *H. americana* can survive for 45 days in rhesus monkeys [7]. The possibility of some degree of visceral development of *H. americana* in primates should not be ruled out. *H. americana* is endemic in U.S states bordering the Gulf of Mexico and the southeastern Atlantic, to the Carolinas. Also notable has been its sporadic recovery from mammals in Kansas [8], Oklahoma [6,9], Tennessee [10], Indiana [11], and Arkansas [6].

The life cycle of *H. americana*, typical for digenetic trematodes of the Family Schistosomatidae, involves a mammalian definitive host in which paired male and female worms are found in mesenteric blood vessels. Eggs are produced, passed in the host's feces, and hatch in water and infect snails of the family Lymnaeidae in this case. Schistosomes reproduce asexually in the snail and release or “shed” many cercariae into water. Cercariae then penetrate the skin of their definitive host and migrate via the lungs to the liver and develop into adult worms.

The only snail species thus far found to be naturally infected with *H. americana* [12] is the small amphibious lymnaeid *Galba cubensis* [13]. It ranges from Florida to Texas with populations known from Oklahoma, Georgia and the Carolinas. Not surprisingly, the historical range of *H. americana* thus appears to correspond with the presence of this snail. Populations of *G. cubensis* also occur in Mexico, South America and the West Indies [14,15]. The cosmopolitan aquatic lymnaeid *Pseudosuccinea columella* [16] is sometimes experimentally susceptible [7] but has not been found naturally infected.

Upon exposure to *H. americana*, dogs experience dermatitis (penetrating cercariae), bloody diarrhea, weight loss, anorexia, vomiting, lethargy, polyuria and polydipsia, and collapse as clinical signs. Granulomatous responses provoked by *H. americana* eggs occur in the liver, intestine, pancreas, and lungs. Similar pathology occurs in raccoons [17]. A distinctive feature of *H. americana* pathology in dogs is hypercalcemia, probably a consequence of granulomatous disease [18] which can lead to renal failure [19,20]. In horses, *H. americana* infection can result in granuloma formation in the lungs, possibly leading to congestive heart failure [21]. Dogs infected with *H. americana* can be treated with praziquantel or fenbendazole. Two consistent messages from veterinarians are that *H. americana* is underdiagnosed and an increasing problem, especially in dogs and horses [22].

Below we provide evidence for the westward expansion of *H. americana*, implicate for the first time a widespread new snail vector *Galba humilis* in natural transmission, provide further details regarding variation within *H. americana* and highlight aspects of the biology of the parasite and its hosts that may both favor spread and complicate control in the future.

## 2 Materials and methods

### 2.1 Collection and screening of field snails

Snails were collected from Mulberry Grove Pond, Moab (Table 1) with handled kitchen strainers swept through aquatic vegetation near the shoreline, or at or above the waterline using forceps. Snails were washed, isolated in individual wells of 24-well plates at mid-day, and left for 2–3 h before screening to identify individual snails shedding cercariae. Snails were re-screened in the early evening (6-8 pm) and the following morning. Snails not shedding cercariae were maintained in the laboratory and re-examined at two-week intervals. *H. americana* cercariae were photographed, preserved in 100% ethanol, or used to infect mice.

**Table 1:** Museum vouchers, locality information, and GenBank Accession numbers of parasites and snails collected as a part of this study. The Louisiana isolate of *H. americana* and several of the *G. humilis* (and other lymnaeid) isolates are part of the existing collections of the Parasite Division, Museum of Southwestern Biology. Detailed information for each specimen is on the Arctos Database (http://arctos.database.museum/SpecimenSearch.cfm).

				GenBank Accession numbers	
Species	Museum Voucher	Locality	Source of Host or Parasite	*cox1*	*ITS*
*Heterobilharzia americana*	MSB:Para:19286	Louisiana	Adult worm from raccoon	MZ020157	–
	MSB:Para:18951	Indiana	Adult worm from raccoon	–	–
	MSB:Para:31807	Moab, Utah	Eggs from raccoon feces	MW425690	MW425378
	MSB:Para:31790	Moab, Utah	Adults from experimental infection	–	–
	MSB:Para:31806	Moab, Utah	Cercariae from field-derived snails	MW963185	–
	MSB:Para:31995	Austin, Texas	Eggs from dog feces	MW963186, MW963187	–
*Galba humilis*	MSB:Host:24243	Moab, Utah	Field-derived snails	MW425684-MW425688	MW427222-MW427227
	MSB:Host:24242	Moab, Utah	Lab-reared snail	MW879390	MW879712
	MSB:Host:22266	Lake Winnibigoshish, Minnesota	Field-derived snail	MW879391	–
	MSB:Host:22819	Tingley Beach, New Mexico	Field-derived snail	MW879392	–
	MSB:Host:23338	Eagles Nest, New Mexico	Field-derived snail	MW879394	–
	MSB:Host:23347	Eagles Nest, New Mexico	Field-derived snail	–	–
	MSB:Host:23371	Valles Caldera, New Mexico	Field-derived snail	MW879393	–
*Galba schirazensis*	MSB:Host:23332	Valle Escondito, New Mexico	Field-derived snail	MW879397	–
*Galba* sp.	MSB:Host:20433	Bosque del Apache, New Mexico	Field-derived snail	MW879396	MW879710
	MSB:Host:20480	Bosque del Apache, New Mexico	Field-derived snail	MW879395	MW879711
*Stagnicola elodes*	MSB:Host:22097	Angel Fire, New Mexico	Field-derived snail	MW879399	
	MSB:Host:24244	Rio Cebolla, New Mexico	Field-derived snail	MW879398	MW879714
	MSB:Host:23703	Pecos Baldy Lake, New Mexico	Field-derived snail	MW879400	MW879713
	MSB:Host:23153	Serpent Lake, New Mexico	Field-derived snail	MW879401	–
	MSB:Host:23349	Black Lake, New Mexico	Field-derived snail	–	MW979408
*Pseudosuccinea columella*	MSB:Host:24245	PetSmart, Albuquerque, New Mexico	Lab-reared snail	–	–
*Procyon lotor* (feces+eggs)	MSB:Host:24240/MSB:Para:31807	Moab, Utah	Raccoon feces and miracidia	MW425353	–
	MSB:Host:24241/MSB:Para:31808	Moab, Utah	Raccoon feces and miracidia	–	–

### 2.2 Establishment and maintenance of a laboratory colony of *Galba humilis*

Mud from Mulberry Grove Pond was placed in plastic dishes (28 cm diameter, 4 cm deep) and sloped to a high point in the center. Spring water was added, leaving a central area uncovered but moist. Snails (20–40 per dish) were fed crushed shrimp pellets and leaf lettuce every other day. Snails were maintained in an ambient light:dark cycle at 25 ± 2 °C. Water was changed regularly. Snails produced egg masses, and progeny were used in experimental infections.

### 2.3 Maintenance of aquatic lymnaeids

Lab-reared *Pseudosuccinea columella* (Albuquerque Pet Smart store), *Stagnicola* cf. *elodes* from Serpent Lake, New Mexico or from Rio Cebolla, Jemez National Recreation Area, New Mexico were maintained in aerated aquaria and fed on leaf lettuce, temperature 25 ± 2 °C.

### 2.4 Examination of fecal samples

At the start of the outbreak, fecal samples from dogs living in the vicinity of Mulberry Grove Pond were collected and sent to the College of Veterinary Medicine and Biomedical Sciences, Texas A&M University, and an established diagnostic PCR assay based on amplification of a portion of the 18S ribosomal gene of *H. americana* [18] was used to determine presence of the parasite. Later, fresh canine, deer or raccoon fecal samples were collected from the ground near the pond; fresh raccoon scats were collected from raccoon latrines. Five grams of fecal material was dispersed in spring water in a 10 cm diameter culture dish, and particles were allowed to settle. The cloudy supernatant was decanted. This process was repeated twice more. The settled material in the dish was examined for the presence of *H. americana* eggs, or swimming miracidia were collected for infection of snails. Some eggs or miracidia were set aside for photography or preserved in 100% ethanol. Identity of the host of origin for positive fecal samples was confirmed as described in the technical appendix. A canine fecal sample from Austin, Texas positive for *H. americana* was later obtained and eggs isolated and stored in 100% ethanol for use in molecular analyses.

### 2.5 Exposure of snails to *H. americana* miracidia

Miracidia were placed in individual wells of 24-well plates, the number varying depending on the trial. A single uninfected lab-reared lymnaeid snail was added to each well and left for three hours. Snails were then maintained as described above. At four weeks post-exposure, and weekly thereafter, exposed snails were isolated and checked for shedding cercariae. Some exposed *G. humilis* and *P. columella* were collected for histological study at 2, 4, 8, or 40 days post-exposure (dpe).

### 2.6 Exposure of mice to *H. americana* cercariae

Cercariae were collected from shedding snails and pooled (numbers depending on the experiment) in beakers containing spring water to a depth of 0.5 cm. A Swiss Webster outbred mouse was placed into each beaker for one hour. Fecal samples from exposed mice were checked weekly starting at 49 dpe for *H. americana* eggs. After 70 dpe, mice were injected with heparin, euthanized using Nembutol [23] and perfused with 199 medium (Sigma-Aldrich, Wisconsin, USA) to collect adult worms. Swimming miracidia for snail exposures were pipetted from water containing homogenized livers of mice with patent infections. Worms were put in 100% ethanol for molecular study or were fixed in 10% buffered formalin for permanent whole mounts [24]. Animal use for this study was approved by the University of New Mexico Institutional Animal Care and Use Committee (IACUC #19-200,813-MC).

### 2.7 Molecular techniques

Because multiple approaches were taken, details of methods for extractions, primers, amplifications, sequencing approaches and phylogenetic analyses of the parasite and snail mitochondrial *cox1* and snail ITS1 datasets are provided in Technical Appendix #1.

## 3 Results

### 3.1 Original outbreak and presumed transmission focus

**Fig. 1:**
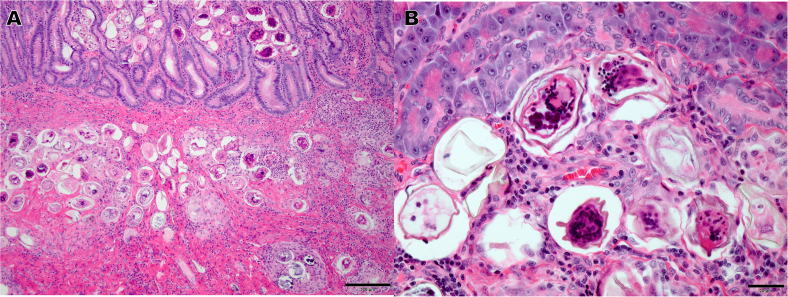
(A) Histological section of intestine of euthanized dog showing large nests of *H. americana* eggs and granulomatous reactions to them, in both the mucosa and submucosa. Scale bar is 100 μm. (B) Histological section of pancreas of euthanized dog showing sections of *H. americana* eggs, some containing developed miracidia. Scale bar is 20 μm.

In fall, 2018, an 8-year-old dog presented with decreased appetite, lethargy, vomiting, diarrhea, polydipsia, and gradual weight loss. The symptoms had begun 3 weeks prior to the other dog in the household being euthanized for similar symptoms. Diagnostic bloodwork revealed azotemia, hypercalcemia, and hyperglobulinemia. One week later, the symptoms had progressed, and diagnostic bloodwork showed renal failure and elevated liver enzymes. The patient was euthanized one week later, and a necropsy performed. On gross examination, the spleen, liver, intestines, kidneys, and pancreas appeared unhealthy and enlarged. Biopsies were submitted for histopathology to Colorado State University. Histopathology revealed mineralization (calcification) of the kidneys. In addition, the intestines and pancreas were infiltrated with trematode eggs resulting in localized tissue inflammation (Fig. 1a,b). All histopathology findings were consistent with schistosomiasis.

**Fig. 2:**
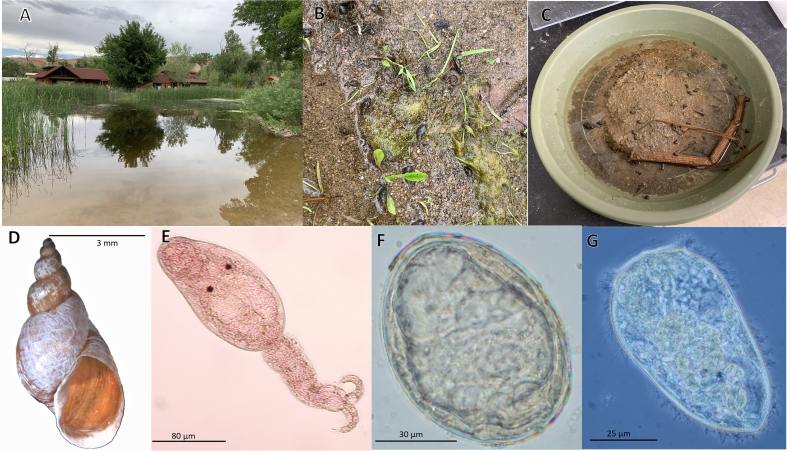
(A) Mulberry Grove Pond in residential area in east-central Moab. (B) Muddy bank of pond, showing several specimens of *G. humilis*. (C) Shallow, flat-bottomed dish containing mud from pond, in which lab-reared populations of *G. humilis* were grown. (D) Shell of *G. humilis*. The largest snails we observed were approximately 11 mm in shell length. (E) Cercaria of *H. americana*. Note the presence of eyespots, an unusual feature for mammalian schistosomes. Scale bar =80 μm. (F) Egg of *H. americana*. Note lack of a spine, and the miracidium within. Scale bar =30 μm. (G) Miracidium of *H. americana*. Scale bar = 50 μm.

The two deceased dogs had lived adjacent to a small man-made irrigation pond (Mulberry Grove Pond) in east-central Moab (Fig. 2a). The pond receives water from nearby Mill Creek and has been filled since at least 2014. We subsequently submitted 12 fecal samples to the Texas A&M Gastrointestinal Laboratory from dogs living in the neighborhood of the pond or that were known to have swum in the pond. Ten of the 12 (83%) were diagnosed positive by PCR for *H. americana*. Some dogs were symptomatic, while others were clinically normal. All dogs were treated with praziquantel and fenbendazole. Symptoms resolved in all treated dogs, and subsequent fecal tests were negative.

### 3.2 Parasitological examinations at mulberry grove pond

**Fig. 3:**
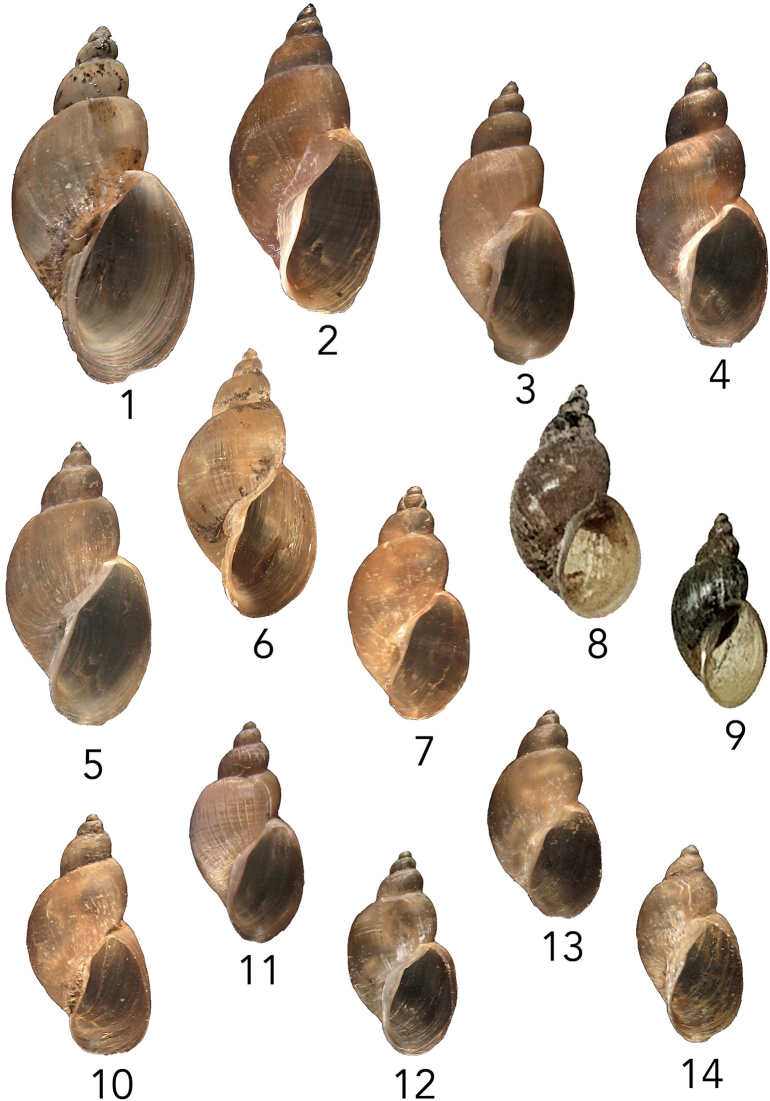
Representatives of *Galba humilis. Galba humilis* from North America H = shell height: 1- Tioga, New York, H = 12.6 mm; 2- Emmet, Michigan, H = 11.0 mm; 3- Beaver Dam, New Mexico, H = 10.3 mm; 4- Campus Lake, Louisiana, H = 10.1 mm; 5- Bizard Island, Montreal, Canada, H = 9.7 mm; 6- Augusta, Virginia, H = 9.7 mm; 7- Philadelphia, Pennsylvania, H = 7.5 mm; 8- Mulberry Grove Pond, Utah, H = 9.6 mm; 9- Mulberry Gove Pond, Utah, H = 5.8 mm; 10- Anderson, Tennessee, H = 6.9 mm; 11- San Antonio, Texas, H = 6.0 mm; 12- Randolph, North Carolina, H = 5.9 mm; 13- Hamilton, Ohio, H = 5.8 mm; 14- Kershaw, South Carolina, H = 5.6 mm.

The pond was visited on multiple occasions to collect either snails or mammalian fecal samples. The pond harbored populations of the aquatic snails *Physa acuta*, *Planorbella trivolvis,* and *Gyraulus parvus*. At the waterline and on the banks of the pond, a population of amphibious snails was found, provisionally identified as *Galba humilis* (Figs. 2b-d, 3) by conchological means [14]. Collections of *G. humilis* made in April 2019 (123 individuals), and July 2019 (25 individuals) were negative for schistosome infections, as were individuals of each of the three aquatic snail species present.

In June 2020, 272 *G. humilis* were collected, three (1.1%) of which shed cercariae (Fig. 2e) consistent with those of *H. americana* [12]. None of the strictly aquatic snail species examined at this time shed schistosome cercariae. Positive *G. humilis* snails kept on an ambient light regime shed cercariae between 6 and 7:30 pm.

Fecal samples from a dog (1), deer (2), and raccoons (9) were collected near the pond. Two of the raccoon scats (2/9, 22.2%) were positive for *H. americana*, one recovered in December 2019, and one in November 2020. The first positive scat was confirmed to be of raccoon origin based on 16S mtDNA sequence; the second has yet to yield amplifiable DNA. (Table 1). Eggs (Fig. 2f) typical of *H. americana* were found, and miracidia (Fig. 2g) hatched from them. Our examinations indicated that transmission of *H. americana* had persisted for at least two years in Moab. Specimens of snails and worms were vouchered at the Museum of Southwestern Biology, Division of Parasites (Table 1).

### 3.3 Experimental infections of snails and mice

Miracidia from the December 2019 raccoon scat were used to expose 26 lab-reared *Pseudosuccinea columella* (10 miracidia per snail), but none became infected. Miracidia from the November 2020 raccoon scat were used to expose 40 lab-reared *G. humilis* (4-8 mm shell length)*,* 4 *Stagnicola elodes* (>10 mm shell length) from Serpent Lake, and 7 *S. elodes* (5- > 10 mm shell length) from Rio Cebolla, all to 5–15 miracidia/snail. Only *G. humilis* became infected and shed cercariae (31 of 40 snails, 77.5%).

**Fig. 4:**
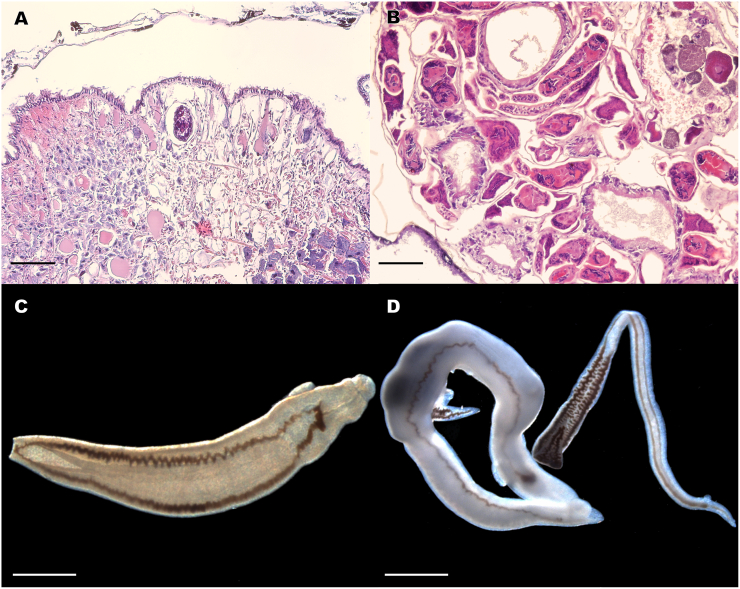
(A) Histological section of *G. humilis* exposed to *H. americana* miracidia, 8 days post exposure. Note presence of mother sporocyst developing in the head foot of the snail. Scale bar = 60 μm. (B) Histological section of *G. humilis* exposed to *H. americana* miracidia, 28 days post-exposure. Note presence of daughter sporocysts harboring developing cercariae in the digestive gland of the snail. Scale bar = 80 μm. (C) Live, unstained adult male of *H. americana* from experimental infection (101 days post-infection), dorsal view, with straight body conformation, testes (white spheres) visible at posterior end. (D) Live, unstained worms, adult male (left) harboring one female within its gynecophoric canal (note bulge containing folded female worm with dark gut and her protruding posterior end) and to the male's right, another female just released from its gynecophoric canal, from infected mouse, 77 days post-infection). Scale bar for adult worms = 500 μm.

Another 23 lab-reared *G. humilis* were later exposed to 1–5 miracidia per snail, and 7 *P. columella* (5-12 mm shell length) were batch-exposed to an undetermined number of miracidia. Only 9 of the *G. humilis* survived, 3 of which were positive (33.3%). Once again, none of the *P. columella* were positive. Histological sections of *G. humilis* exposed to *H. americana* revealed developing schistosome sporocysts (Fig. 4a,b). Infected *G. humilis* were again observed to release cercariae in the early evening hours. Shedding *G. humilis* had shells up to 11 mm long, and 5 of the heavier shedders released an average of 810 (range 740–860) cercariae/snail/shed.

First using cercariae pooled from two naturally infected *G. humilis*, we exposed two mice to infection. At 101 dpe, they were euthanized and perfused and both found to contain only male *H. americana* (Fig. 4c). Using cercariae obtained from experimentally infected *G. humilis*, one mouse was exposed to 100 cercariae pooled from 20 snails and another to 150 cercariae pooled from 15 snails. At 77 dpe, adult schistosomes consistent with *H. americana* males and females were perfused from the portal vein or dissected from the mesenteric veins of both mice. The male photographed initially harbored two females in its gynecophoric canal (Fig. 4d). Numerous miracidia were obtained from the liver and used to infect *G. humilis* snails for subsequent life cycle maintenance.

### 3.4 Molecular confirmation of identifications of *Galba humilis* and *Heterobilharzia americana*

**Fig. 5:**
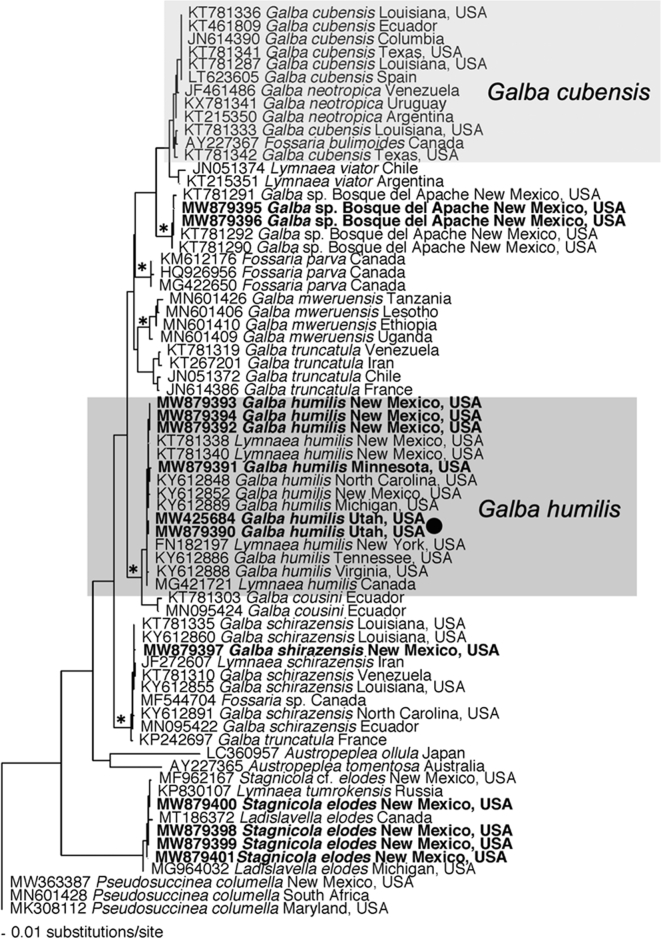
Phylogenetic tree for relevant snails based on Bayesian analysis of *cox1* with nodal support indicated on the branches by posterior probabilities as asterisk for >0.98. The clade for *G. humilis* is outlined in a dark gray box, light gray box are *G. cubensis,* the other species found infected in nature. The samples from this study examined for *Heterobilharzia americana* infections and/or used in experimental exposures are in bold. The relevant sample from Utah is noted with black circle. Additional taxa examined in New Mexico are from the MSB Parasite Division. Taxa are preceded with their corresponding GenBank accession number and followed by their collection locality (U.S. state or country). See Table 1 for accession numbers and MSB numbers.

Snails provisionally identified as *Galba humilis* were confirmed as such based on comparisons with *cox1* sequences from several relevant lymnaeid taxa [13] and from our own collections (Table 1). This comparison groups the Moab specimens with 13 other samples identified as *G. humilis* from different parts of North America (Fig. 3, Fig. 5). Supplementary Fig. 1 is of an ITS1 tree, which includes ITS1 sequences from *G. humilis* from this study.

**Fig. 6:**
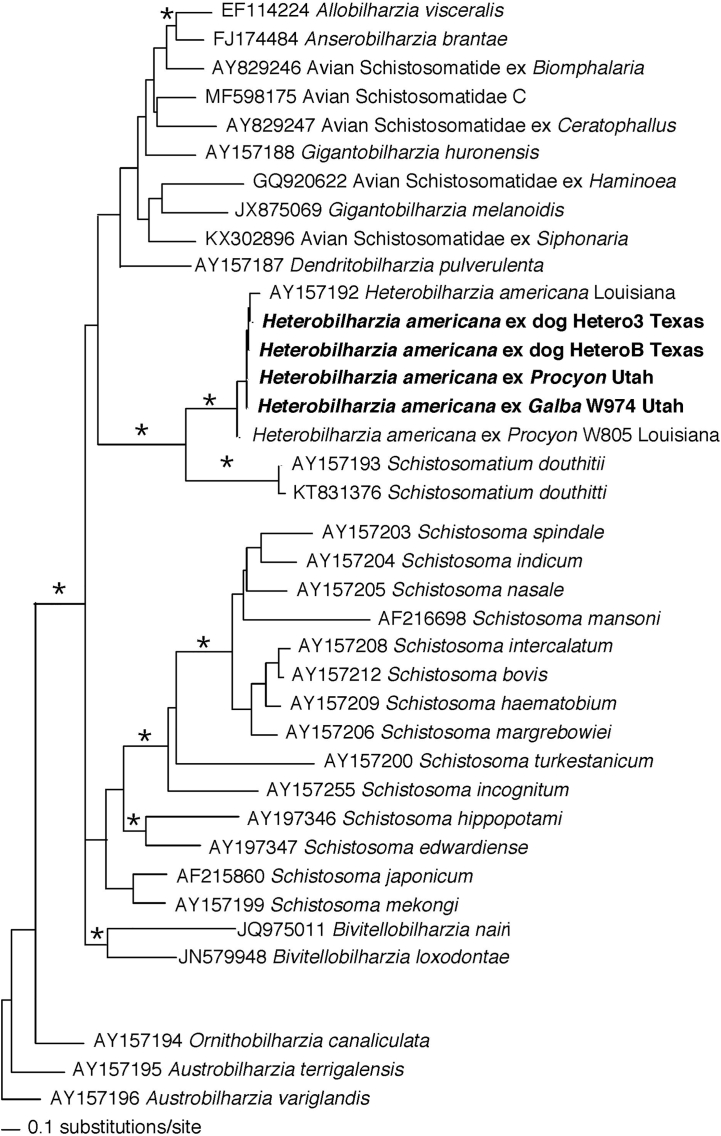
Phylogenetic tree for relevant schistosomes, including *Heterobilharzia americana*, based on Bayesian analysis of *cox1* with nodal support indicated on the branches by posterior probabilities as asterisk for >0.98. The samples from this study are in bold and outlined in a gray box. Taxa names are preceded by their corresponding GenBank accession number and followed by their collection locality (U.S. state). See Table 1 for accession numbers and MSB numbers.

For *H. americana* from Utah, *cox1* sequences derived from eggs and miracidia obtained from the 2019 raccoon scat sample and cercariae from experimentally-infected *G. humilis* in 2020 showed uncorrected *p*-distances of 0% with *H. americana* from Texas, 4% with *H. americana* from Louisiana, and ~ 20% with *Schistosomatium douthitti*, the monospecific sister genus of North American mammalian schistosome (Fig. 6).

### 3.5 Phylogenetic considerations for *Galba humilis* and *Heterobilharzia americana*

*Galba humilis* from Moab groups with high support with other representatives of this species from widely separated parts of North America (Fig. 3, Fig. 5). It also groups in a clade separate from that of the other two known host species of *H. americana*, *G. cubensis,* and *P. columella*.

The close relationships between *H. americana* and *S. douthitti* is confirmed (Fig. 6). The results also revealed isolates of *H. americana* from Utah and Texas to be more similar to each other than to an isolate from Louisiana.

## 4 Discussion

The appearance of *H. americana* in Utah marks the most westerly report for this endemic North American schistosome. Coupled with the Indiana report [11], this parasite has either had a wider distribution than heretofore appreciated or is currently expanding its range. Northward range expansion of *G. cubensis* could play a role in range expansion of the schistosome, and this snail has been reported as far north as Oklahoma, but the infected snails recovered in Utah were not *G. cubensis*, but *G. humilis*. It also seems unlikely that the semi-tropical species *G. cubensis* could have been involved in transmission of *H. americana* in Indiana [11].

Malek [[24], [25]] noted that *G. humilis* from Michigan was marginally susceptible (0.7–1.6% positive) to experimental infection with *H. americana*. Insofar as the Moab isolate of *G. humilis* was 77.5% positive following experimental exposure, and we noted, as have others [26], that *P. columella* was not compatible, it seems likely that the spread of *H. americana* has been facilitated by acquiring increased compatibility with *G. humilis*. *G. humilis* occurs widely in Utah and North America. It also serves as an intermediate host for *Fasciola hepatica* [27].

*Galba humilis* is one of at least 40 related subspecies and species of “fossarine” lymnaeids, all relatively small (<15 mm in shell height), living at or above the waterline but at times submersed as well [14,28]. Many of these taxa will eventually be synonymized [13]. Collectively their range covers much of North America, many Caribbean islands, and parts of Central and South America. Although their occurrence tends to be spotty, they can be locally very abundant [28]. Susceptibility to *H. americana* of fossarine lymnaeids from most of these areas is unknown. Our phylogenetic analysis showing other species of *Galba* as close relatives to *G. cubensis* and *G. humilis* suggests the possibility they too could potentially be infected by *H. americana*.

The fact that cercariae are released from snails at night is likely an indication of the extent to which this parasite is adapted to raccoons which commonly forage in and around aquatic habitats at night [29]. Raccoons (see https://upload.wikimedia.org/wikipedia/commons/0/04/Raccoon-range.png for range map) were not common in Utah through the 20th century [30], but as with other locations throughout the American west, they have since become much more abundant, particularly in urban areas. Elimination of predators, expanding agriculture and urbanization, and climate-related increases in available food all favor the spread of raccoons [31]. A recent climate modeling analysis suggested raccoons will expand their range northward but may diminish in numbers in more tropical areas of their known range [32,33]. This may help to explain the apparent absence of *H. americana* from areas south of Texas given that a permissive snail host, *G. cubensis,* is known from Mexico, South America, and Cuba. Alternatively, this apparent absence may reflect a lack of survey information.

Recovery of *H. americana* from Utah and Indiana suggests it is not precluded from colder climates. Its closest relative, *S. douthitti*, occurs as far north as Alaska [34]. The establishment of the mostly tropical species *Schistosoma haematobium* in Corsica has prompted the suggestion that schistosomes are “pre-adapted” to colder climates [35].

Infected dogs from endemic areas may also be critical in introducing *H. americana* into new habitats (possibly including Moab). Parasites carried in dog feces may infect local snail populations, which in turn might permit spread of infection to local raccoons, which are probably better maintenance hosts. Construction of man-made habitats like Mulberry Grove Pond also favor *H. americana* by attracting dogs and raccoons to these new snail-supporting habitats [36].

Our sequencing results suggest there might be strain- or possibly species-level differences within *H. americana*, a supposition already discussed by others [37,38]. We found a Louisiana isolate from raccoons differed by as much as 4.1% in *cox1* sequences from our Utah and Texas isolates, values approaching those often considered to be typical of species delineations [[39], [40], [41]]. Our results also suggest that *H. americana* from Moab may have originated from Texas. Further studies are needed to learn if the isolates from different states have diverged sufficiently to be considered different species.

## 5 Conclusions

If raccoons or dogs infected with *H. americana* colonize areas where susceptible amphibious lymnaeids like *G. humilis* are common, then the spread of *H. americana* in North America will likely continue. Any mammals using such habitats would be at risk, potentially suffering symptoms ranging from dermatitis to granulomatous disease as a consequence. The role of raccoons in the transmission of *H. americana* adds to the health concerns they already pose as hosts for rabies and the intestinal roundworm *Baylisascaris procyonis* [42]. Introductions of raccoons into Europe have already led to emergence of *B. procyonis* there and increases the likelihood that *H. americana* could also extend its range to Europe. Amphibious lymnaeids such as *Galba truncatula* are common in Eurasia and might prove to be susceptible to *H. americana*. Additional study of possible incorporation of new snail hosts and range shifts are clearly warranted for *H. americana*, an emerging schistosome capable of infecting a broad range of mammals with potentially severe pathological consequences. Furthermore, the difficulties involved in controlling raccoons, or snails colonizing muddy banks of an increasing numbers of man-made aquatic habitats, will prove to be challenging, just as are efforts to control human schistosomiasis.

**The following are the supplementary data related to this article.Supplementary Fig. 1:**
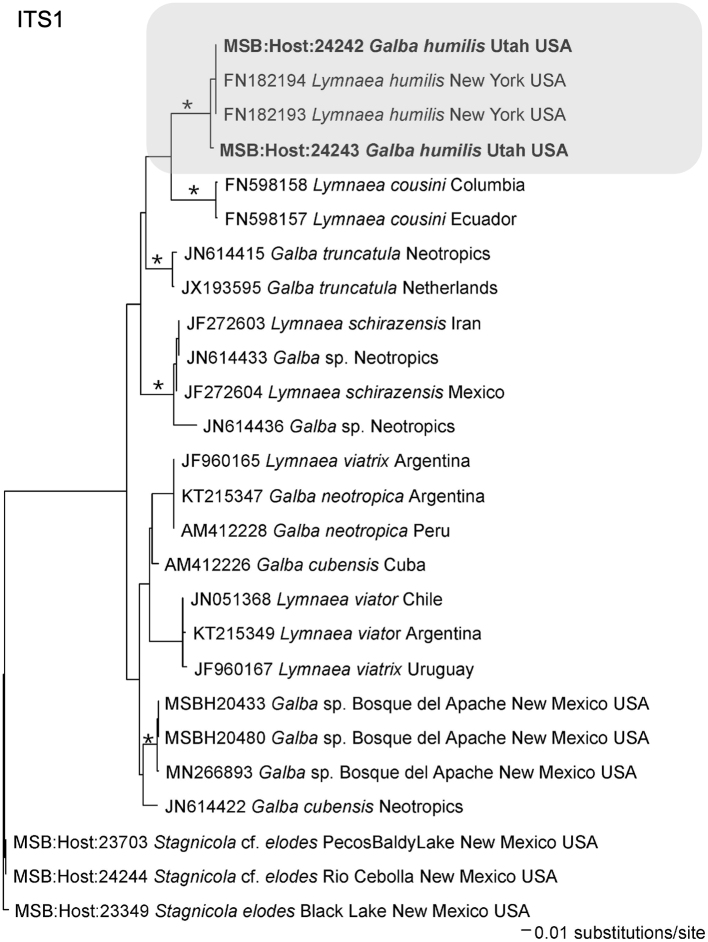
Phylogenetic tree for *Galba humilis* based on Bayesian analysis of ITS1 with nodal support indicated on the branches by posterior probabilities as asterisk for >0.98. The clade for *G. humilis* is outlined in a light gray box. See Table 1 for accession numbers and MSB numbers. Samples in bold are part of this study.

Supplementary material Image 1

## Funding

This work was supported by the core laboratory support provided by The 10.13039/100000002National Institutes of Health [R37AI101438] and technical assistance at the University of New Mexico Molecular Biology Facility was supported by the National Institute of General Medical Sciences of the National Institutes of Health [P30GM110907]. The content for this paper is solely the responsibility of the authors and does not necessarily represent the official views of the National Institutes of Health.

## Declaration of Competing Interest

None.
