# Supplementary material for: An outbreak of canine schistosomiasis in Utah: Acquisition of a new snail host (Galba humilis) by Heterobilharzia americana, a pathogenic parasite on the move
Source: One Health. 2021 Jun 17;13:100280. doi: 10.1016/j.onehlt.2021.100280 (PMC8254006; doi:10.1016/j.onehlt.2021.100280)
Supplement: Supplementary file 1 — Supplementary material [file mmc2.docx]

**Technical appendix 1:**

*Parasite DNA Extraction*

DNA from miracidia was extracted using the Qiagen DNeasy Blood and Tissue kit (Qiagen, Valencia, California) with a modified protocol for small volume extractions [1]. DNA from worm fragments or cercariae was extracted with the QIAamp DNA Micro Kit according to manufacturer’s guidelines, except samples were eluted with 30 ul of buffer.

*Fecal sample DNA Extraction*

A small amount of fecal material was preserved in ethanol and then DNA was extracted using the standard protocol of the Qiagen DNeasy Blood and Tissue kit.

*Snail DNA Extraction*

Snail tissue from MSB:Host:24242 and fecal material were extracted with the standard Qiagen DNeasy Blood and Tissue kit protocol. For the remaining snails, a small piece of tissue was taken from the head foot of individual snails. DNA was extracted using the E.Z.N.A. Mollusc DNA kit (Omega Biotek) following the manufacturer’s protocol.

*Sequencing*

PCR amplification of multiple loci was performed on snail, parasite, and host tissues, using a variety of primer sets. Parasite 18S: 18SJVSQF, 18SmodF, 18SJVSQR; snail ITS1: Lim1657, ITS1RIXO, parasite ITS2: 3SN, BD2; snail ITS2: NEWS2, ITS2-RIXO; parasite 28S: C1, D2, 28SJVSQF, LSU3; raccoon scat, parasite, and snail 16S mtDNA: 16Sar, 16Sbr; parasite cox1 mtDNA: Cox1_Schisto_5, Cox1_Schisto_3, COIF15, COIR15; and snail cox1: LCO1490, HCO2198). For these primers see Alda et al., 2021 [2], Brant and Loker 2009 [3], DeJong et al. 2001 [4], Folmer et al., 1994 [5], Jothikumar et al. 2015 [6] and Lockyer et al., 2003 [10]. These reactions used 10 μl Taq 2X MeanGreen Master Mix (Empirical Biosciences) resulting in 0.2 mM dNTPs, 1.5 mM MgCl2, 0.5 μM each primer, in a total volume of 20 μl. In some cases, DNA was amplified by using the TaKara Ex Taq kit (Takara Biomedicals, Otsu, Japan). Thermocycling conditions were (with exceptions see below) 94 °C for 2 min; 35 cycles of 95°C for 15s, 48.5°C for 1 min, 72°C for 1 min, and a final extension at 72 °C for 7 min. Thermocycling conditions on an Eppendorf MasterCycler for parasite *cox1* Cox1_Schisto_5, Cox1_Schisto_3 were 94 °C for 6 min; 3 cycles for each annealing temp 51-47°C then 20 cycles 46°C with denaturation 94°C for 30s and extension 72°C for 2 min, and a final extension at 72 °C for 5 min. Thermocycling conditions on an Eppendorf MasterCycler for snail ITS (Lim1657, ITS1RIXO) were as follows: 94 °C for 6 min; 3 cycles for each annealing temp 60-56°C then 20 cycles 55°C with denaturation 94°C for 30s and extension 72°C for 2 min, and a final extension at 72 °C for 5 min.

PCR products were visualized on 1.0% TBE agarose gels stained with SYBR′′ Safe (Invitrogen) or GelRed® (Biotium, Fremont, CA). Successful PCR amplicons were enzymatically purified using ExoSAP-IT (Affymetrix, Santa Clara, CA) or E.Z.N.A® Cycle Pure Kit (Omega Bio-Tek, Norcross, GA). Purified PCR products were sequenced using the Applied Biosystems BigDye direct sequencing kit, version 3.1 (Applied Biosystems, Foster City, CA, USA). Sanger DNA sequencing was completed at the Genomic Sciences Laboratory at North Carolina State University, Raleigh, NC, USA, or at the University of New Mexico. Chromatograms were edited in Sequencher v 5.0 (Gene Codes Corporation, Ann Arbor, MI, USA) and sequences were aligned by eye in Se-Al v 2.0a11 (tree.- bio.ed.ac.uk).

*Phylogenetic Analyses*

Phylogenetic analyses of the mitochondrial *cox1* for the parasite and the snail were performed using Bayesian inference in MrBayes [12] with default priors for *cox1* (parameters un-linked so each partition by codon has its own set of parameters; Nst = 6 rates-invgamma). Model selection was estimated using ModelTest. Partitions by codon evolved under different rates (preset applyto = (all) ratepr = variable). Model selection was estimated using ModelTest [13]. Four chains were run simultaneously for 5 × 105 generations, the first 5000 trees with preasymptotic likelihood scores were discarded as burn-in, and the retained trees were used to generate 50% majority-rule consensus trees and posterior probabilities. Phylogenetic analysis of the snail *ITS1* was performed by Neighbor Joining. Outgroups used have been defined in previous analyses [2].

**References**

1. B.L. Webster, Isolation and preservation of schistosome eggs and larvae in RNAlater^(R)^ facilitates genetic profiling of individuals, Parasites & vectors. 2 (2009), pp. 50-50. DOI: 10.1186/1756-3305-2-50
2. P. Alda, M. Lounnas, A.A. Vazquez, R. Ayaqui, M. Calvopina, M. Celi-Erazo, R.T. Dillon, Jr., L.C. Gonzalez Ramirez, E.S. Loker, J. Muzzio-Aroca, A.O. Narvaez, O. Noya, A.E. Pereira, L.M. Robles, R. Rodriguez-Hidalgo, N. Uribe, P. David, P. Jarne, J.P. Pointier, S. Hurtrez-Bousses, Systematics and geographical distribution of *Galba* species, a group of cryptic and worldwide freshwater snails, Mol Phylogenet Evol. 157 (2021), pp. 107035. DOI: 10.1016/j.ympev.2020.107035
3. S.V. Brant, E.S. Loker, Schistosomes in the southwest United States and their potential for causing cercarial dermatitis or ‘swimmer's itch’, Journal of Helminthology. 83 (2009), pp. 191-198. DOI: 10.1017/S0022149X09308020
4. R.J. DeJong, J.A.T. Morgan, W.L. Paraense, J.P. Pointier, M. Amarista, P.F.K. Ayeh-Kumi, A. Babiker, C.S. Barbosa, P. Bremond, A.P. Canese, C.P. de Souza, C. Dominguez, S. File, A. Gutierrez, R.N. Incani, T. Kawano, F. Kazibwe, J. Kpikpi, N.J.S. Lwambo, R. Mimpfoundi, F. Njiokou, J.N. Poda, M. Sene, L.E. Velasquez, M. Yong, C.M. Adema, B.V. Hofkin, G.M. Mkoji, E.S. Loker, Evolutionary relationships and biogeography of *Biomphalaria* (Gastropoda: Planorbidae) with implications regarding its role as host of the human bloodfluke, *Schistosoma* *mansoni*, Molecular Biology and Evolution. 18 (2001), pp. 2225-2239. DOI 10.1093/oxfordjournals.molbev.a003769
5. O. Folmer, M. Black, W. Hoeh, R. Lutz, R. Vrijenhoek, DNA primers for amplification of mitochondrial cytochrome c oxidase subunit I from diverse metazoan invertebrates, Molecular Marine Biology and Biotechnology. 3 (1994), pp. 294-299.
6. N. Jothikumar, B.J. Mull, S.V. Brant, E.S. Loker, J. Collinson, W.E. Secor, V.R. Hill, Real-time PCR and sequencing assays for rapid detection and identification of avian schistosomes in environmental samples, Appl Environ Microbiol. 81 (2015), pp. 4207-4215. DOI: 10.1128/AEM.00750-15
7. J. Bowles, D.P. McManus, Rapid discrimination of *Echinococcus* species and strains using a polymerase chain reaction-based RFLP method, Molecular and Biochemical Parasitology. 57 (1993), pp. 231-239. DOI: https://doi.org/10.1016/0166-6851(93)90199-8
8. J. Bowles, D.P. McManus, A molecular phylogeny of the human schistosomes, Molecular Phylogenetics and Evolution 4 (1995) pp. 103-109
9. Dvorak J, Vanacova S, Hampl V, Flegr J, Horak P. Comparison of European *Trichobilharzia* species based on ITS1 and ITS2 sequences, Parasitology. 124 (2002), pp. 307-313. DOI: 10.1017/S0031182001001238
10. A.E. Lockyer, P.D. Olson, P. Ostergaard, D. Rollinson, D.A. Johnston, S.W. Attwood, V.R. Southgate, P. Horak, S.D. Snyder, T.H. Le, T. Agatsuma, D.P. McManus, A.C. Carmichael, S. Naem, D.T. Littlewood, The phylogeny of the Schistosomatidae based on three genes with emphasis on the interrelationships of *Schistosoma* Weinland, 1858, Parasitology. 126 (2003), pp. 203-224. DOI: 10.1017/s0031182002002792
11. S.V. Brant, J.A. Morgan, G.M. Mkoji, S.D. Snyder, R.P. Rajapakse, E.S. Loker, An approach to revealing blood fluke life cycles, taxonomy, and diversity: provision of key reference data including DNA sequence from single life cycle stages, J Parasitol. 92 (2006), pp. 77-88. DOI: 10.1645/ge-3515.1

[12] J.P. Huelsenbeck, F. Ronquist, MRBAYES: Bayesian inference of phylogenetic trees, Bioinformatics. 17 (2001), pp. 754-755. DOI: 10.1093/bioinformatics/17.8.754

[13] D. Posada, K.A. Crandall, MODELTEST: testing the model of DNA substitution, Bioinformatics. 14 (1998), pp. 817-818. DOI: 10.1093/bioinformatics/14.9.817
